# Aqua­(dicyanamido-κ*N* ^1^)(nitrato-κ^2^ *O*,*O*′)(2,3,5,6-tetra-2-pyridylpyrazine-κ^3^ *N* ^2^,*N* ^1^,*N* ^6^)manganese(II)

**DOI:** 10.1107/S1600536808041755

**Published:** 2008-12-13

**Authors:** Lorena Callejo, Noelia De la Pinta, Pablo Vitoria, Roberto Cortés

**Affiliations:** a Departamento de Química Inorgánica, Facultad de Ciencia y Tecnología, Universidad del País Vasco, Apdo. 644, E-48080 Bilbao, Spain; b Departamento de Química Inorgánica, Facultad de Farmacia, Universidad del País Vasco, Apdo. 450, E-01080 Vitoria, Spain

## Abstract

In the title compound, [Mn(C_2_N_3_)(NO_3_)(C_24_H_16_N_6_)(H_2_O)], the central manganese(II) ion is hepta­coordinated to a tridentate 2,3,5,6-tetra-2-pyridylpyrazine ligand (tppz), a bidentate nitrate ligand, a terminal monodentate dicyanamide ligand (dca) and a water mol­ecule. The structure contains isolated neutral complexes, which are linked by O(water)—H⋯N hydrogen bonds generating chains along [010].

## Related literature

For related structures containing coordination compounds with the ligands tppz and dca, see: Carranza *et al.* (2003 ▶); Hsu *et al.* (2005 ▶). For related literature, see: Lainé *et al.* (1995 ▶).
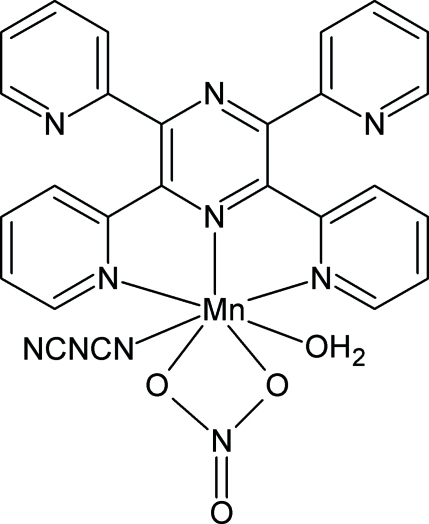


## Experimental

### 

#### Crystal data

[Mn(C_2_N_3_)(NO_3_)(C_24_H_16_N_6_)(H_2_O)]
*M* *_r_* = 589.44
Monoclinic, 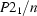

*a* = 14.0988 (11) Å
*b* = 9.7739 (8) Å
*c* = 18.7205 (13) Å
β = 94.491 (6)°
*V* = 2571.8 (3) Å^3^
*Z* = 4
Mo *K*α radiation
μ = 0.57 mm^−1^
*T* = 298 (2) K
0.42 × 0.31 × 0.08 mm

#### Data collection

Oxford Diffraction Xcalibur 2 diffractometer
Absorption correction: analytical (*CrysAlis RED*; Oxford Diffraction, 2007 ▶) *T*_min_ = 0.856, *T*_max_ = 0.969
24694 measured reflections
7480 independent reflections
4848 reflections with *I* > 2σ(*I*)
*R*_int_ = 0.054

#### Refinement

*R*[*F* ^2^ > 2σ(*F* ^2^)] = 0.049
*wR*(*F* ^2^) = 0.119
*S* = 0.93
7480 reflections
376 parameters
2 restraints
H atoms treated by a mixture of independent and constrained refinement
Δρ_max_ = 0.77 e Å^−3^
Δρ_min_ = −0.31 e Å^−3^

### 

Data collection: *CrysAlis CCD* (Oxford Diffraction, 2007 ▶); cell refinement: *CrysAlis RED* (Oxford Diffraction, 2007 ▶); data reduction: *CrysAlis RED*; program(s) used to solve structure: *SIR2004* (Burla *et al.*, 2005 ▶); program(s) used to refine structure: *SHELXL97* (Sheldrick, 2008 ▶); molecular graphics: *DIAMOND* (Brandenburg, 2007 ▶); software used to prepare material for publication: *WinGX* (Farrugia, 1999 ▶) and *PLATON* (Spek, 2003 ▶).

## Supplementary Material

Crystal structure: contains datablocks I, global. DOI: 10.1107/S1600536808041755/fj2176sup1.cif

Structure factors: contains datablocks I. DOI: 10.1107/S1600536808041755/fj2176Isup2.hkl

Additional supplementary materials:  crystallographic information; 3D view; checkCIF report

## Figures and Tables

**Table 1:** Selected bond lengths (Å)

Mn1—O1*W*	2.1537 (15)
Mn1—N7	2.2457 (18)
Mn1—O1	2.2648 (15)
Mn1—N2	2.2796 (15)
Mn1—N1	2.3015 (15)
Mn1—N3	2.3247 (16)
Mn1—O2	2.4021 (15)

**Table 2:** Hydrogen-bond geometry (Å, °)

*D*—H⋯*A*	*D*—H	H⋯*A*	*D*⋯*A*	*D*—H⋯*A*
O1*W*—H1*W*⋯N6^i^	0.78 (2)	2.03 (2)	2.800 (2)	174 (3)
O1*W*—H2*W*⋯N7^i^	0.80 (2)	2.24 (2)	3.029 (2)	168 (2)

Symmetry code: (i) 
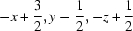
.

**Fig. 1.**
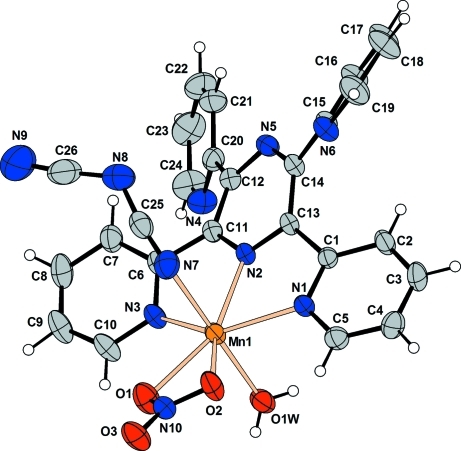
The molecular structure of (I), with atom labels and 50% probability displacement ellipsoids for non-H atoms.

**Fig. 2.**
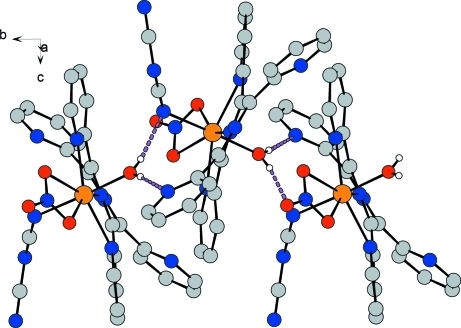
Detail of the chain generated by the O(water)—H···N hydrogen bonds.

## supplementary crystallographic information

### Comment

Only a few examples are known of coordination compounds with the ligands dicyanamido (dca) and 2,3,5,6-tetra-2-pyridylpyrazine (tppz) (Carranza *et al.*, 2003; Hsu *et al.*, 2005).

The molecule of the title compound (I) (Fig. 1) contains a central manganese(II) metal heptacoordinated to a terminal dicyanamide ligand, three nitrogen atoms of the tppz ligand, two oxygen atoms of the nitrate group, and one water molecule.

The central pyrazine ring of the tppz is severely distorted from planarity (N2—C11—C12—N5 = 20.9 (3)°, N2—C13—C14—N5 = 19.9 (2)°) and adopts a twist-boat conformation with a puckering amplitude of 0.215 (2)Å (Spek, 2003). The pyridyl rings are rotated away from planarity with the pyrazine ring, with angles between planes of 25.3 (1) and 21.5 (1)° for the ones coordinated to Mn(II), and larger [31.1 (1), 35.9 (1)°] for the other ones.

The O(water)—H···N hydrogen bonds formed between the water as donor, and a non-coordinated pyridyl ring and the coordinated nitrogen atom of dca as acceptors, generate chains of molecules along the [010] direction (Fig.2).

### Experimental

The title compound was prepared by mixing two acetonitrile solutions (10 ml each) of Mn(NO_3_)_2_.4H_2_O (125.5 mg, 0.50 mmol) and 2,3,5,6-tetrakis(2-pyridyl)pirazine (97.1 mg, 0.25 mmol). After vigorous stirring for 3 h at a temperature of 30°C, a yellow precipitate appeared. To the resulting solution, a water/acetonitrile (50%) solution (10 ml) of sodium dicyanamide was added, and it was stirred at 40°C for 3 h, and then 2 days at room temperature. The precipitate was filtered off and yellow plaques formed from the resulting solution by slow evaporation at room temperature.

### Refinement

H atoms bonded to O atoms were located in a difference map and refined with distance restraints of O—H = 0.82 (2), and with *U*_iso_(H) = 1.5*U*_eq_(O). Other H atoms were positioned geometrically and refined using a riding model, with C—H = 0.93 Å and with *U*_iso_(H) = 1.2 times *U*_eq_(C).

### Crystal data

**Table:**

[Mn(C_2_N_3_)(NO_3_)(C_24_H_16_N_6_)(H_2_O)]	*F*(000) = 1204
*M**_r_* = 589.44	*D*_x_ = 1.522 Mg m^−^^3^*D*_m_ = 1.475 Mg m^−^^3^*D*_m_ measured by flotation
Monoclinic, *P*2_1_/*n*	Mo *K*α radiation, λ = 0.71073 Å
Hall symbol: -P 2yn	Cell parameters from 2543 reflections
*a* = 14.0988 (11) Å	θ = 3.2–31.9°
*b* = 9.7739 (8) Å	µ = 0.57 mm^−^^1^
*c* = 18.7205 (13) Å	*T* = 298 K
β = 94.491 (6)°	Prism, yellow
*V* = 2571.8 (3) Å^3^	0.42 × 0.31 × 0.08 mm
*Z* = 4	

### Data collection

**Table:**

Oxford Diffraction Xcalibur 2 diffractometer	7480 independent reflections
Radiation source: Enhance (Mo) X-ray Source	4848 reflections with *I* > 2σ(*I*)
graphite	*R*_int_ = 0.054
Detector resolution: 8.3504 pixels mm^-1^	θ_max_ = 30.0°, θ_min_ = 2.7°
ω scans	*h* = −18→19
Absorption correction: analytical (*CrysAlis RED*; Oxford Diffraction, 2007)	*k* = −13→12
*T*_min_ = 0.856, *T*_max_ = 0.969	*l* = −26→26
24694 measured reflections	

### Refinement

**Table:**

Refinement on *F*^2^	Primary atom site location: structure-invariant direct methods
Least-squares matrix: full	Secondary atom site location: difference Fourier map
*R*[*F*^2^ > 2σ(*F*^2^)] = 0.049	Hydrogen site location: difference Fourier map
*wR*(*F*^2^) = 0.119	H atoms treated by a mixture of independent and constrained refinement
*S* = 0.93	*w* = 1/[σ^2^(*F*_o_^2^) + (0.0676*P*)^2^] where *P* = (*F*_o_^2^ + 2*F*_c_^2^)/3
7480 reflections	(Δ/σ)_max_ = 0.003
376 parameters	Δρ_max_ = 0.77 e Å^−^^3^
2 restraints	Δρ_min_ = −0.31 e Å^−^^3^

### Special details

**Table:**

Experimental. CrysAlis RED (Oxford Diffraction Ltd., 2007) Analytical numeric absorption correction using a multifaceted crystal model.
Geometry. All s.u.'s (except the s.u. in the dihedral angle between two l.s. planes) are estimated using the full covariance matrix. The cell s.u.'s are taken into account individually in the estimation of s.u.'s in distances, angles and torsion angles; correlations between s.u.'s in cell parameters are only used when they are defined by crystal symmetry. An approximate (isotropic) treatment of cell s.u.'s is used for estimating s.u.'s involving l.s. planes.

### Fractional atomic coordinates and isotropic or equivalent isotropic displacement parameters (Å^2^)

**Table:**

	*x*	*y*	*z*	*U*_iso_*/*U*_eq_	
Mn1	0.666558 (19)	0.45676 (3)	0.206427 (15)	0.02617 (9)	
N1	0.78906 (11)	0.48216 (15)	0.29414 (8)	0.0258 (3)	
N2	0.80556 (10)	0.38970 (15)	0.16256 (8)	0.0244 (3)	
N3	0.63611 (11)	0.34999 (16)	0.09605 (9)	0.0302 (4)	
O1	0.51063 (11)	0.51437 (17)	0.19225 (9)	0.0447 (4)	
O2	0.58673 (10)	0.60852 (16)	0.28292 (8)	0.0424 (4)	
O1W	0.63075 (10)	0.27874 (16)	0.26656 (9)	0.0386 (4)	
H1W	0.5843 (14)	0.239 (3)	0.2725 (15)	0.058*	
H2W	0.6758 (15)	0.237 (2)	0.2837 (13)	0.058*	
O3	0.44256 (10)	0.67307 (17)	0.25070 (9)	0.0483 (4)	
N4	0.84607 (13)	0.11733 (18)	0.00078 (9)	0.0397 (4)	
N5	0.97237 (11)	0.37317 (17)	0.10010 (8)	0.0298 (4)	
N6	1.03910 (11)	0.63452 (17)	0.22266 (9)	0.0348 (4)	
N7	0.70245 (13)	0.64756 (18)	0.14738 (10)	0.0399 (4)	
N8	0.77072 (15)	0.6846 (3)	0.03399 (12)	0.0622 (6)	
N9	0.6902 (2)	0.7210 (4)	−0.08341 (14)	0.1065 (12)	
N10	0.51142 (11)	0.60058 (17)	0.24260 (9)	0.0321 (4)	
C1	0.87743 (13)	0.45325 (18)	0.27688 (10)	0.0242 (4)	
C2	0.95391 (14)	0.4433 (2)	0.32828 (11)	0.0325 (4)	
H2	1.0141	0.4195	0.3153	0.039*	
C3	0.93869 (15)	0.4693 (2)	0.39852 (11)	0.0377 (5)	
H3	0.9889	0.4644	0.4337	0.045*	
C4	0.84871 (15)	0.5027 (2)	0.41657 (11)	0.0360 (5)	
H4	0.8373	0.5222	0.4638	0.043*	
C5	0.77617 (14)	0.5066 (2)	0.36298 (11)	0.0315 (4)	
H5	0.7151	0.5272	0.3753	0.038*	
C6	0.71035 (13)	0.33917 (19)	0.05521 (10)	0.0271 (4)	
C7	0.69805 (15)	0.3326 (2)	−0.01871 (11)	0.0374 (5)	
H7	0.7505	0.3313	−0.0459	0.045*	
C8	0.60688 (17)	0.3280 (2)	−0.05189 (12)	0.0472 (6)	
H8	0.5971	0.3241	−0.1016	0.057*	
C9	0.53124 (16)	0.3294 (2)	−0.01012 (13)	0.0470 (6)	
H9	0.4694	0.3213	−0.0309	0.056*	
C10	0.54801 (14)	0.3427 (2)	0.06286 (12)	0.0394 (5)	
H10	0.4962	0.3470	0.0906	0.047*	
C11	0.80572 (13)	0.34520 (18)	0.09524 (9)	0.0253 (4)	
C12	0.89402 (13)	0.31643 (19)	0.06821 (10)	0.0281 (4)	
C13	0.88645 (12)	0.42660 (18)	0.19952 (10)	0.0242 (4)	
C14	0.96892 (13)	0.43718 (19)	0.16272 (10)	0.0266 (4)	
C15	1.05445 (13)	0.5179 (2)	0.18756 (10)	0.0292 (4)	
C16	1.14420 (14)	0.4738 (2)	0.17324 (12)	0.0371 (5)	
H16	1.1523	0.3931	0.1480	0.045*	
C17	1.22185 (15)	0.5528 (3)	0.19744 (14)	0.0480 (6)	
H17	1.2832	0.5262	0.1887	0.058*	
C18	1.20685 (16)	0.6706 (3)	0.23433 (14)	0.0530 (6)	
H18	1.2580	0.7242	0.2519	0.064*	
C19	1.11509 (16)	0.7090 (2)	0.24518 (13)	0.0459 (6)	
H19	1.1056	0.7905	0.2693	0.055*	
C20	0.90828 (14)	0.2209 (2)	0.00846 (10)	0.0304 (4)	
C21	0.98469 (15)	0.2352 (2)	−0.03278 (11)	0.0406 (5)	
H21	1.0265	0.3085	−0.0260	0.049*	
C22	0.99719 (19)	0.1369 (3)	−0.08467 (12)	0.0523 (6)	
H22	1.0479	0.1432	−0.1134	0.063*	
C23	0.9341 (2)	0.0306 (3)	−0.09314 (13)	0.0547 (7)	
H23	0.9412	−0.0367	−0.1275	0.066*	
C24	0.85993 (19)	0.0258 (2)	−0.04955 (14)	0.0517 (6)	
H24	0.8169	−0.0462	−0.0558	0.062*	
C25	0.72974 (15)	0.6673 (2)	0.09222 (13)	0.0368 (5)	
C26	0.72404 (19)	0.7046 (3)	−0.02745 (15)	0.0559 (7)	

### Atomic displacement parameters (Å^2^)

**Table:**

	*U*^11^	*U*^22^	*U*^33^	*U*^12^	*U*^13^	*U*^23^
Mn1	0.02005 (14)	0.03028 (16)	0.02787 (16)	0.00049 (11)	−0.00010 (10)	−0.00116 (12)
N1	0.0219 (8)	0.0282 (8)	0.0271 (8)	0.0000 (6)	−0.0003 (6)	−0.0003 (6)
N2	0.0204 (7)	0.0269 (8)	0.0251 (8)	0.0003 (6)	−0.0021 (6)	−0.0009 (6)
N3	0.0219 (8)	0.0328 (9)	0.0350 (9)	−0.0007 (6)	−0.0036 (7)	−0.0055 (7)
O1	0.0369 (9)	0.0503 (9)	0.0458 (9)	0.0057 (7)	−0.0047 (7)	−0.0149 (8)
O2	0.0284 (8)	0.0533 (10)	0.0436 (9)	0.0041 (7)	−0.0096 (7)	−0.0050 (8)
O1W	0.0255 (8)	0.0370 (9)	0.0528 (9)	−0.0031 (6)	−0.0008 (7)	0.0092 (7)
O3	0.0277 (8)	0.0557 (10)	0.0618 (10)	0.0143 (7)	0.0046 (7)	−0.0050 (8)
N4	0.0423 (10)	0.0398 (10)	0.0380 (10)	−0.0039 (8)	0.0098 (8)	−0.0084 (8)
N5	0.0228 (8)	0.0358 (9)	0.0307 (8)	−0.0010 (6)	0.0025 (7)	−0.0006 (7)
N6	0.0246 (8)	0.0384 (10)	0.0410 (10)	−0.0014 (7)	−0.0009 (7)	−0.0049 (8)
N7	0.0442 (11)	0.0323 (10)	0.0425 (11)	0.0007 (8)	−0.0020 (9)	0.0041 (8)
N8	0.0456 (12)	0.0891 (18)	0.0531 (13)	−0.0092 (12)	0.0118 (11)	0.0062 (12)
N9	0.085 (2)	0.184 (4)	0.0492 (16)	−0.030 (2)	−0.0023 (15)	0.0184 (19)
N10	0.0250 (8)	0.0374 (9)	0.0340 (9)	0.0026 (7)	0.0023 (7)	0.0033 (8)
C1	0.0232 (9)	0.0247 (9)	0.0244 (9)	−0.0010 (7)	−0.0008 (7)	0.0012 (7)
C2	0.0222 (9)	0.0421 (12)	0.0322 (10)	0.0045 (8)	−0.0039 (8)	−0.0009 (9)
C3	0.0352 (11)	0.0465 (13)	0.0293 (10)	0.0023 (9)	−0.0104 (9)	−0.0015 (9)
C4	0.0433 (12)	0.0399 (11)	0.0244 (10)	0.0016 (9)	0.0001 (9)	−0.0035 (8)
C5	0.0298 (10)	0.0349 (10)	0.0301 (10)	0.0015 (8)	0.0044 (8)	−0.0026 (8)
C6	0.0243 (9)	0.0260 (9)	0.0299 (10)	−0.0001 (7)	−0.0042 (8)	−0.0031 (8)
C7	0.0347 (11)	0.0454 (12)	0.0310 (11)	0.0026 (9)	−0.0036 (9)	−0.0008 (9)
C8	0.0478 (14)	0.0553 (15)	0.0355 (12)	0.0057 (11)	−0.0164 (10)	−0.0070 (11)
C9	0.0319 (11)	0.0535 (14)	0.0526 (14)	0.0025 (10)	−0.0161 (10)	−0.0121 (12)
C10	0.0231 (10)	0.0444 (13)	0.0497 (13)	−0.0008 (9)	−0.0051 (9)	−0.0131 (10)
C11	0.0236 (9)	0.0261 (9)	0.0257 (9)	0.0001 (7)	−0.0014 (7)	0.0000 (7)
C12	0.0260 (9)	0.0314 (10)	0.0266 (9)	0.0000 (8)	0.0008 (8)	0.0014 (8)
C13	0.0198 (8)	0.0256 (9)	0.0266 (9)	0.0025 (7)	−0.0025 (7)	−0.0007 (7)
C14	0.0205 (9)	0.0298 (10)	0.0290 (9)	0.0016 (7)	−0.0014 (7)	−0.0006 (8)
C15	0.0204 (9)	0.0357 (11)	0.0310 (10)	−0.0017 (7)	−0.0009 (7)	0.0017 (8)
C16	0.0233 (10)	0.0424 (12)	0.0456 (12)	0.0007 (8)	0.0028 (9)	−0.0035 (10)
C17	0.0205 (10)	0.0605 (15)	0.0624 (16)	−0.0012 (10)	−0.0005 (10)	−0.0021 (13)
C18	0.0262 (11)	0.0605 (16)	0.0708 (17)	−0.0119 (11)	−0.0050 (11)	−0.0108 (14)
C19	0.0358 (12)	0.0441 (13)	0.0565 (14)	−0.0066 (10)	−0.0036 (11)	−0.0125 (11)
C20	0.0305 (10)	0.0349 (11)	0.0257 (9)	0.0040 (8)	0.0021 (8)	0.0001 (8)
C21	0.0350 (11)	0.0513 (13)	0.0367 (12)	0.0006 (10)	0.0103 (9)	−0.0003 (10)
C22	0.0533 (15)	0.0690 (18)	0.0374 (13)	0.0108 (13)	0.0209 (11)	−0.0010 (12)
C23	0.0729 (19)	0.0531 (16)	0.0396 (13)	0.0094 (13)	0.0147 (13)	−0.0128 (11)
C24	0.0624 (17)	0.0437 (14)	0.0500 (14)	−0.0076 (11)	0.0112 (12)	−0.0135 (11)
C25	0.0302 (11)	0.0321 (11)	0.0464 (13)	−0.0029 (8)	−0.0076 (10)	0.0002 (10)
C26	0.0520 (16)	0.0694 (18)	0.0481 (15)	−0.0163 (13)	0.0146 (13)	−0.0007 (13)

### Geometric parameters (Å, °)

**Table:**

Mn1—O1W	2.1537 (15)	C3—H3	0.9300
Mn1—N7	2.2457 (18)	C4—C5	1.376 (3)
Mn1—O1	2.2648 (15)	C4—H4	0.9300
Mn1—N2	2.2796 (15)	C5—H5	0.9300
Mn1—N1	2.3015 (15)	C6—C7	1.383 (3)
Mn1—N3	2.3247 (16)	C6—C11	1.488 (2)
Mn1—O2	2.4021 (15)	C7—C8	1.384 (3)
N1—C5	1.337 (2)	C7—H7	0.9300
N1—C1	1.341 (2)	C8—C9	1.371 (3)
N2—C11	1.333 (2)	C8—H8	0.9300
N2—C13	1.336 (2)	C9—C10	1.374 (3)
N3—C10	1.346 (2)	C9—H9	0.9300
N3—C6	1.348 (2)	C10—H10	0.9300
O1—N10	1.264 (2)	C11—C12	1.408 (3)
O2—N10	1.256 (2)	C12—C20	1.483 (3)
O1W—H1W	0.776 (16)	C13—C14	1.401 (3)
O1W—H2W	0.800 (16)	C14—C15	1.485 (3)
O3—N10	1.221 (2)	C15—C16	1.383 (3)
N4—C24	1.325 (3)	C16—C17	1.387 (3)
N4—C20	1.340 (3)	C16—H16	0.9300
N5—C14	1.333 (2)	C17—C18	1.367 (3)
N5—C12	1.334 (2)	C17—H17	0.9300
N6—C19	1.336 (3)	C18—C19	1.377 (3)
N6—C15	1.342 (3)	C18—H18	0.9300
N7—C25	1.146 (3)	C19—H19	0.9300
N8—C25	1.284 (3)	C20—C21	1.381 (3)
N8—C26	1.294 (4)	C21—C22	1.387 (3)
N9—C26	1.128 (4)	C21—H21	0.9300
C1—C2	1.391 (2)	C22—C23	1.369 (4)
C1—C13	1.487 (2)	C22—H22	0.9300
C2—C3	1.373 (3)	C23—C24	1.377 (3)
C2—H2	0.9300	C23—H23	0.9300
C3—C4	1.377 (3)	C24—H24	0.9300
			
O1W—Mn1—N7	177.74 (7)	N3—C6—C11	114.99 (16)
O1W—Mn1—O1	89.83 (6)	C7—C6—C11	122.91 (18)
N7—Mn1—O1	89.42 (7)	C6—C7—C8	119.3 (2)
O1W—Mn1—N2	101.50 (6)	C6—C7—H7	120.3
N7—Mn1—N2	80.11 (6)	C8—C7—H7	120.3
O1—Mn1—N2	152.04 (6)	C9—C8—C7	118.7 (2)
O1W—Mn1—N1	84.71 (6)	C9—C8—H8	120.6
N7—Mn1—N1	94.37 (6)	C7—C8—H8	120.6
O1—Mn1—N1	136.40 (6)	C8—C9—C10	119.1 (2)
N2—Mn1—N1	70.72 (5)	C8—C9—H9	120.4
O1W—Mn1—N3	93.87 (6)	C10—C9—H9	120.4
N7—Mn1—N3	88.18 (6)	N3—C10—C9	123.0 (2)
O1—Mn1—N3	84.12 (6)	N3—C10—H10	118.5
N2—Mn1—N3	69.81 (5)	C9—C10—H10	118.5
N1—Mn1—N3	139.34 (6)	N2—C11—C12	118.14 (16)
O1W—Mn1—O2	92.71 (6)	N2—C11—C6	114.86 (16)
N7—Mn1—O2	85.11 (6)	C12—C11—C6	126.90 (17)
O1—Mn1—O2	54.58 (5)	N5—C12—C11	118.71 (17)
N2—Mn1—O2	148.05 (5)	N5—C12—C20	116.28 (16)
N1—Mn1—O2	82.45 (5)	C11—C12—C20	124.94 (17)
N3—Mn1—O2	138.12 (5)	N2—C13—C14	118.22 (16)
C5—N1—C1	117.95 (16)	N2—C13—C1	114.65 (16)
C5—N1—Mn1	123.77 (13)	C14—C13—C1	127.13 (16)
C1—N1—Mn1	117.69 (12)	N5—C14—C13	119.12 (16)
C11—N2—C13	120.85 (16)	N5—C14—C15	116.12 (16)
C11—N2—Mn1	119.97 (11)	C13—C14—C15	124.75 (17)
C13—N2—Mn1	117.31 (12)	N6—C15—C16	123.04 (18)
C10—N3—C6	117.61 (17)	N6—C15—C14	116.53 (17)
C10—N3—Mn1	122.22 (13)	C16—C15—C14	120.42 (18)
C6—N3—Mn1	116.22 (12)	C15—C16—C17	118.3 (2)
N10—O1—Mn1	97.27 (11)	C15—C16—H16	120.9
N10—O2—Mn1	90.98 (11)	C17—C16—H16	120.9
Mn1—O1W—H1W	135 (2)	C18—C17—C16	119.0 (2)
Mn1—O1W—H2W	114.3 (19)	C18—C17—H17	120.5
H1W—O1W—H2W	110 (3)	C16—C17—H17	120.5
C24—N4—C20	117.06 (19)	C17—C18—C19	119.2 (2)
C14—N5—C12	120.17 (16)	C17—C18—H18	120.4
C19—N6—C15	117.48 (18)	C19—C18—H18	120.4
C25—N7—Mn1	133.43 (17)	N6—C19—C18	123.0 (2)
C25—N8—C26	122.9 (2)	N6—C19—H19	118.5
O3—N10—O2	122.18 (17)	C18—C19—H19	118.5
O3—N10—O1	121.26 (17)	N4—C20—C21	123.30 (19)
O2—N10—O1	116.56 (16)	N4—C20—C12	115.50 (17)
N1—C1—C2	122.17 (17)	C21—C20—C12	121.08 (18)
N1—C1—C13	115.05 (15)	C20—C21—C22	118.0 (2)
C2—C1—C13	122.73 (17)	C20—C21—H21	121.0
C3—C2—C1	118.60 (18)	C22—C21—H21	121.0
C3—C2—H2	120.7	C23—C22—C21	119.3 (2)
C1—C2—H2	120.7	C23—C22—H22	120.3
C2—C3—C4	119.67 (19)	C21—C22—H22	120.3
C2—C3—H3	120.2	C22—C23—C24	118.3 (2)
C4—C3—H3	120.2	C22—C23—H23	120.9
C5—C4—C3	118.29 (19)	C24—C23—H23	120.9
C5—C4—H4	120.9	N4—C24—C23	124.0 (2)
C3—C4—H4	120.9	N4—C24—H24	118.0
N1—C5—C4	123.26 (18)	C23—C24—H24	118.0
N1—C5—H5	118.4	N7—C25—N8	172.7 (2)
C4—C5—H5	118.4	N9—C26—N8	174.4 (3)
N3—C6—C7	121.95 (17)		
			
O1W—Mn1—N1—C5	−63.95 (15)	Mn1—N1—C5—C4	170.63 (15)
N7—Mn1—N1—C5	113.98 (16)	C3—C4—C5—N1	−1.4 (3)
O1—Mn1—N1—C5	20.22 (19)	C10—N3—C6—C7	5.5 (3)
N2—Mn1—N1—C5	−168.10 (16)	Mn1—N3—C6—C7	−152.74 (16)
N3—Mn1—N1—C5	−153.72 (14)	C10—N3—C6—C11	−178.84 (17)
O2—Mn1—N1—C5	29.50 (15)	Mn1—N3—C6—C11	23.0 (2)
O1W—Mn1—N1—C1	107.06 (13)	N3—C6—C7—C8	−4.3 (3)
N7—Mn1—N1—C1	−75.01 (14)	C11—C6—C7—C8	−179.65 (19)
O1—Mn1—N1—C1	−168.77 (12)	C6—C7—C8—C9	−0.4 (3)
N2—Mn1—N1—C1	2.91 (12)	C7—C8—C9—C10	3.5 (4)
N3—Mn1—N1—C1	17.29 (17)	C6—N3—C10—C9	−2.1 (3)
O2—Mn1—N1—C1	−159.49 (13)	Mn1—N3—C10—C9	154.67 (18)
O1W—Mn1—N2—C11	100.10 (14)	C8—C9—C10—N3	−2.4 (4)
N7—Mn1—N2—C11	−81.53 (14)	C13—N2—C11—C12	10.4 (3)
O1—Mn1—N2—C11	−12.0 (2)	Mn1—N2—C11—C12	174.40 (13)
N1—Mn1—N2—C11	−179.74 (15)	C13—N2—C11—C6	−166.21 (16)
N3—Mn1—N2—C11	10.18 (13)	Mn1—N2—C11—C6	−2.2 (2)
O2—Mn1—N2—C11	−145.24 (13)	N3—C6—C11—N2	−13.9 (2)
O1W—Mn1—N2—C13	−95.37 (13)	C7—C6—C11—N2	161.74 (19)
N7—Mn1—N2—C13	83.01 (13)	N3—C6—C11—C12	169.79 (18)
O1—Mn1—N2—C13	152.51 (13)	C7—C6—C11—C12	−14.5 (3)
N1—Mn1—N2—C13	−15.21 (12)	C14—N5—C12—C11	10.7 (3)
N3—Mn1—N2—C13	174.72 (14)	C14—N5—C12—C20	−166.23 (17)
O2—Mn1—N2—C13	19.30 (19)	N2—C11—C12—N5	−20.9 (3)
O1W—Mn1—N3—C10	84.37 (16)	C6—C11—C12—N5	155.22 (18)
N7—Mn1—N3—C10	−94.67 (16)	N2—C11—C12—C20	155.68 (18)
O1—Mn1—N3—C10	−5.06 (16)	C6—C11—C12—C20	−28.1 (3)
N2—Mn1—N3—C10	−174.79 (17)	C11—N2—C13—C14	9.5 (3)
N1—Mn1—N3—C10	170.75 (14)	Mn1—N2—C13—C14	−154.90 (13)
O2—Mn1—N3—C10	−14.0 (2)	C11—N2—C13—C1	−170.97 (16)
O1W—Mn1—N3—C6	−118.53 (13)	Mn1—N2—C13—C1	24.63 (19)
N7—Mn1—N3—C6	62.43 (14)	N1—C1—C13—N2	−21.5 (2)
O1—Mn1—N3—C6	152.04 (14)	C2—C1—C13—N2	155.81 (17)
N2—Mn1—N3—C6	−17.70 (13)	N1—C1—C13—C14	157.98 (18)
N1—Mn1—N3—C6	−32.16 (17)	C2—C1—C13—C14	−24.7 (3)
O2—Mn1—N3—C6	143.05 (12)	C12—N5—C14—C13	9.4 (3)
O1W—Mn1—O1—N10	97.99 (12)	C12—N5—C14—C15	−169.43 (17)
N7—Mn1—O1—N10	−79.87 (13)	N2—C13—C14—N5	−19.9 (3)
N2—Mn1—O1—N10	−147.21 (12)	C1—C13—C14—N5	160.60 (17)
N1—Mn1—O1—N10	15.86 (16)	N2—C13—C14—C15	158.84 (17)
N3—Mn1—O1—N10	−168.10 (12)	C1—C13—C14—C15	−20.6 (3)
O2—Mn1—O1—N10	4.54 (10)	C19—N6—C15—C16	−0.9 (3)
O1W—Mn1—O2—N10	−92.39 (11)	C19—N6—C15—C14	−179.71 (19)
N7—Mn1—O2—N10	88.24 (11)	N5—C14—C15—N6	144.16 (18)
O1—Mn1—O2—N10	−4.53 (10)	C13—C14—C15—N6	−34.6 (3)
N2—Mn1—O2—N10	150.67 (11)	N5—C14—C15—C16	−34.7 (3)
N1—Mn1—O2—N10	−176.69 (11)	C13—C14—C15—C16	146.5 (2)
N3—Mn1—O2—N10	6.46 (15)	N6—C15—C16—C17	1.1 (3)
O1—Mn1—N7—C25	−110.2 (2)	C14—C15—C16—C17	179.9 (2)
N2—Mn1—N7—C25	43.7 (2)	C15—C16—C17—C18	0.0 (4)
N1—Mn1—N7—C25	113.3 (2)	C16—C17—C18—C19	−1.3 (4)
N3—Mn1—N7—C25	−26.1 (2)	C15—N6—C19—C18	−0.5 (4)
O2—Mn1—N7—C25	−164.7 (2)	C17—C18—C19—N6	1.6 (4)
Mn1—O2—N10—O3	−171.75 (17)	C24—N4—C20—C21	−0.4 (3)
Mn1—O2—N10—O1	7.42 (17)	C24—N4—C20—C12	−176.33 (19)
Mn1—O1—N10—O3	171.25 (16)	N5—C12—C20—N4	147.61 (18)
Mn1—O1—N10—O2	−7.93 (18)	C11—C12—C20—N4	−29.1 (3)
C5—N1—C1—C2	2.4 (3)	N5—C12—C20—C21	−28.5 (3)
Mn1—N1—C1—C2	−169.16 (14)	C11—C12—C20—C21	154.8 (2)
C5—N1—C1—C13	179.72 (16)	N4—C20—C21—C22	0.0 (3)
Mn1—N1—C1—C13	8.2 (2)	C12—C20—C21—C22	175.8 (2)
N1—C1—C2—C3	−2.6 (3)	C20—C21—C22—C23	0.0 (4)
C13—C1—C2—C3	−179.76 (18)	C21—C22—C23—C24	0.3 (4)
C1—C2—C3—C4	0.8 (3)	C20—N4—C24—C23	0.7 (4)
C2—C3—C4—C5	1.1 (3)	C22—C23—C24—N4	−0.6 (4)
C1—N1—C5—C4	−0.4 (3)		

### Hydrogen-bond geometry (Å, °)

**Table:**

*D*—H···*A*	*D*—H	H···*A*	*D*···*A*	*D*—H···*A*
O1W—H1W···N6^i^	0.78 (2)	2.03 (2)	2.800 (2)	174 (3)
O1W—H2W···N7^i^	0.80 (2)	2.24 (2)	3.029 (2)	168 (2)
C5—H5···O2	0.93	2.53	3.122 (2)	122
C7—H7···N4	0.93	2.60	2.966 (3)	104
C10—H10···O1	0.93	2.51	3.027 (3)	116

Symmetry codes: (i) −*x*+3/2, *y*−1/2, −*z*+1/2.
